# Magnetic Iron Oxide Nanoparticles for Multimodal Imaging and Therapy of Cancer

**DOI:** 10.3390/ijms140815910

**Published:** 2013-07-31

**Authors:** Reju Thomas, In-Kyu Park, Yong Yeon Jeong

**Affiliations:** 1 Department of Radiology, Chonnam National University Hwasun Hospital, Chonnam National University Medical School, Gwangju 501-746, Korea; E-Mail: regeth@gmail.com; 2 Department of Biomedical Science, Research Institute of Medical Sciences, Chonnam National University Medical School, Gwangju 501-746, Korea; E-Mail: pik96d@gmail.com

**Keywords:** SPION, multimodal, multifunctional, theranostics, MRI, CT, SPECT, PET

## Abstract

Superparamagnetic iron oxide nanoparticles (SPION) have emerged as an MRI contrast agent for tumor imaging due to their efficacy and safety. Their utility has been proven in clinical applications with a series of marketed SPION-based contrast agents. Extensive research has been performed to study various strategies that could improve SPION by tailoring the surface chemistry and by applying additional therapeutic functionality. Research into the dual-modal contrast uses of SPION has developed because these applications can save time and effort by reducing the number of imaging sessions. In addition to multimodal strategies, efforts have been made to develop multifunctional nanoparticles that carry both diagnostic and therapeutic cargos specifically for cancer. This review provides an overview of recent advances in multimodality imaging agents and focuses on iron oxide based nanoparticles and their theranostic applications for cancer. Furthermore, we discuss the physiochemical properties and compare different synthesis methods of SPION for the development of multimodal contrast agents.

## 1. Introduction

Early detection and treatment of cancer are critical factors for a favorable prognosis. Cancer diagnosis using nanotechnology is an emerging field. Tremendous efforts in biomedical research have been devoted to improving the sensitivity and accuracy of diagnosing cancer using early detection methods and to improving the efficacy of treatment methods. A non-invasive diagnosis of patients can be achieved using *in vivo* imaging techniques. These techniques are powerful tools for visualizing the abnormal state of the body and for monitoring biological situations at the target site. Examples of non-invasive imaging techniques include the following: computed tomography (CT), magnetic resonance imaging (MRI), nuclear imaging of positron emission tomography (PET), single-photon emission computed tomography (SPECT), and optical (or fluorescence) imaging. These imaging techniques have provided scientists and clinicians with the ability to acquire *in vivo* images of anatomy and physiology in both animals and humans [1–6]. MRI uses non-ionizing radiation and is an integral tool in clinical radiology and experimental settings. MRI is very effective in characterizing soft tissue and has a high tissue resolution. Additionally, the modality is tomographic with unlimited penetration into tissues [4,7].

Advances in nanotechnology have permitted new possibilities for theranostics, which are defined as the combination of therapy and imaging within a single platform [8,9]. Nanotechnology is applied to molecular imaging in the form of imaging probes capable of enhancing the sensitivity of the image and the specificity toward the target tissue. Usually, the imaging probe consists of nanoparticles conjugated with active targeting ligands [10,11]. Other types of tumor targeting are based on passive targeting mediated by enhanced permeability and retention (EPR) [12,13]. Superparamagnetic iron oxide nanoparticles (SPION) have a superparamagnetic iron core, which makes them useful as T2 contrast agents for MRI. SPION can be detected with high sensitivity, and both the iron and polymer components of SPION are biocompatible and degradable [14].

The size of iron oxide nanoparticles plays a major role in target cell uptake and elimination from the body. Spleen and liver capture nanoparticles of more than 200 nm in diameter whereas particles having sizes below 10 nm are selectively filtered by renal systems and eliminated from body [15]. The surface properties of nanoparticles can be modified to accommodate targeting [16]. With the recent interest in multimodal imaging, nanotechnology has focused on developing novel probes that combine various agents to form new all-in-one probes with both multimodal capability and therapeutic functionality [17]. Multifunctional nanoparticles enable multimodal imaging with the combination of two or more imaging modalities or theranostics for simultaneous imaging and therapy. These techniques are expected to overcome the hurdles of traditional diagnosis and therapy through optimized therapy called “personalized medicine”[6]. The majority of nanoparticles in development include drug conjugates and complexes, micelles, dendrimers, vesicles, core–shell particles, microbubbles, and carbon nanotubes [18]. Among these nanoparticles, magnetic nanoparticles exhibit not only unique material properties but also an integrated design capacity for cell targeting, imaging, and therapy. This combination of properties makes nanoparticles ideal platform materials for theranostics. In addition, magnetic nanoparticles have been proven to serve as workhorses for multimodality preclinical imaging as they allow MRI, nuclear and optical imaging [15,19]. In this review, we provide an overview of recent advances in multimodality imaging, focusing on iron oxide based nanoparticles and on their theranostic applications for cancer.

## 2. Synthesis of Iron Oxide Nanoparticles

SPION are used as an MRI contrast agent because the T2 relaxivity of a SPION-based agent is much higher than that of gadolinium agents. The physiochemical properties of SPION, such as charge, size, and surface chemistry, can influence biodistribution, stability, and metabolism [20]. By giving proper surface coating that are biocompatible and biodegradable, SPION can avoid immune response and serum protein adsorption. Surface charge is a major factor in determining the colloidal nature of nanoparticles, and it can change size of nanoparticles by aggregation. Hence neutral surfaces are more biocompatible than charged surfaces. Surface charge can also influence plasma protein binding that directly affects *in vivo* biodistribution and nanoparticle clearance from the body [21].

A typical SPION is composed of magnetite (Fe_3_O_4_) or maghemite (Fe_2_O_3_), with appropriate coatings to maintain aqueous stability. The SPION are synthesized by a wide range of methods, including co-precipitation, thermal decomposition, and microemulsion (Table 1). There are also recently discovered methods, such as microwave assisted synthesis, the hydrolysis method, and microfluidic synthesis [22–24]. Thermal decomposition for the synthesis of SPION is generally more popular as the nanoparticles obtained this way possess high crystallinity, high magnetization, and a narrow size distribution. Simultaneously, the nanoparticles are hydrophobic, which is required to provide appropriate coating for biomedical application [25]. The co-precipitation method is the most commonly accepted method for synthesizing SPION. It involves the addition of a concentrated base to divalent or trivalent ferrous salt solutions. The resulting nanoparticles are polydispersed, with a size range from 2–15 nm [15,26]. The particles aggregate and grow, thereby decreasing the surface free energy [27]. The microemulsion technique has the advantage of controlling the size of nanoparticles by acting as a nano-reactor. This technique involves the formation of SPION either by water-in-oil or oil-in-water emulsion. The SPION formed have a controlled size distribution between 2 and 12 nm [15].

Superparamagnetism is attributed to the single domain nature of the nanoparticle, which has a net magnetic dipole. In a magnetic field, the magnetic domains of nanoparticles re-orient themselves in a manner similar to paramagnetic materials, but the magnetic moment of nanoparticles will be much higher than that of paramagnetic substances. In the absence of a magnetic field, the dipole randomly orients with zero magnetic moment. Due to this property, SPION have less chance of aggregation [15]. The function of SPION in MRI contrast enhancement is attributed to their ability to change the nuclear spin relaxation of water protons and cause the region of interest to darken.

It is very important to understand the toxic effect and the mechanism by which iron oxide nanoparticles can cause toxicity before using them for clinical application. Iron oxide nanoparticles are converted to elemental iron species, and finally merged to our body reserves or utilised for the formation of haemoglobin. Biosafety of iron oxide nanoparticle (Fe3O_4_ and MnFe_3_O_4_) have been established up to 200 μg/mL concentration [16]. But when cells are exposed to very high dose of iron oxide nanoparticles, formation of excess ROS takes place that affects normal functioning of the cell, leading to apoptosis or cell death [21].

## 3. Imaging Techniques

Imaging techniques used in the clinic generally include MRI, optical imaging, CT, and PET or SPECT (Table 2). Each imaging technique has its own unique advantages and intrinsic limitations, such as insufficient sensitivity or spatial resolution, which make it difficult to obtain accurate and reliable information at the disease site [19].

MRI is a useful problem-solving diagnostic tool in the clinical field because it has higher spatial resolution and contrast in soft tissue than other imaging modalities. MRI is based on the magnetism property of protons that align themselves in a very large magnetic field. These protons originate from water molecules present in our body tissue. A radiofrequency generated at a particular frequency, known as the “resonance frequency,” can flip the spin of a proton. When the electromagnetic field is turned off, the proton flips back to the original state, generating a radiofrequency signal. This process is called “relaxation.” The receiver coils measure this relaxation, which is turned into an image by a computer algorithm [29]. MRI contrast agents are used to modify the relaxation rates at time T1 or T2. T1 contrast agents, such as gadolinium chelators, enhance the positive signal on T1-weighted images, while T2 agents, such as SPION-based contrast agents, decrease the signal intensity on T2-weighted images.

Optical imaging techniques have used different physical parameters of light to interact with tissue, and a number of different optical imaging approaches have been described. These techniques rely on fluorescence, absorption, reflectance, or bioluminescence as a source of contrast. Optical imaging consists mostly of NIRF (near-infrared fluorescence), reflectance imaging, and bioluminescence imaging. Despite its relatively recent emergence, optical imaging is rapidly being implemented in various clinical and research areas. The advantages of optical imaging are that most of the optical contrast agents are non-toxic and comparatively inexpensive, versatile, and sensitive [30–32]. Optical imaging involves an optical contrast agent, either organic or inorganic, that can fluoresce at various excitation wavelengths. Early optical imaging was used in cancer diagnosis based on the variation in endogenous fluorescence of neoplastic tissue. However, due to the difficulty in distinguishing the diagnostic signal component from background fluorescence, exogenous contrast agents were developed [31]. Nanoparticle based optical contrast agents, such as “quantum dots,” were thus developed to have superior imaging properties compared to organic counterparts. The major issues encountered by optical imaging are the inability to quantify the image and the autofluorescence of normal tissue against the fluorescence of the contrast agent, which can severely impair the image quality [13].

CT is another imaging technique widely used in the clinic for diagnosis of various diseases. It uses x-rays to obtain images through slices of the body area. The main advantage of the CT imaging technique is that it produces images with high spatial resolution. The most popular CT contrast agents are iodine-based compounds. These agents work by blocking X-rays, thereby providing contrast and enhancing a part of the body. Iodine-based contrast agents produce side effects, such as vomiting, itching, and anaphylactic shock. Among non-ionic and ionic contrast agents, ionic agents prove to be more harmful, especially for patients with renal problems [33–35]. Therefore, recent research has focused on developing a gold nanoparticle based contrast agent for CT. Gold nanoparticles are biocompatible and are capable of targeting the tumor by the EPR effect. This material has a very high X-ray absorption coefficient, which makes it a suitable agent for replacing iodine in CT imaging. Gold is a metal with a high atomic number and is therefore considered a strong candidate for CT imaging because it provides better x-ray attenuation and contrast [36].

PET and SPECT fall under the category of nuclear imaging because they are based on the detection of radioisotopes that emit one or two gamma rays or positrons. Both are excellent imaging techniques due to their specificity, sensitivity, and fast detection time. However, both techniques lack the ability to display good spatial resolution. PET uses isotopes such as ^68^Ga, ^76^Br, ^94m^Tc, ^11^C, ^13^N, ^15^O, ^18^F, and ^64^Cu. SPECT uses gamma-emitting heavy radioisotopes such as ^123^I, ^99m^Tc, and ^133^Xe [37].

## 4. Multimodal Strategies for Cancer Theranostics

**Figure 1:**
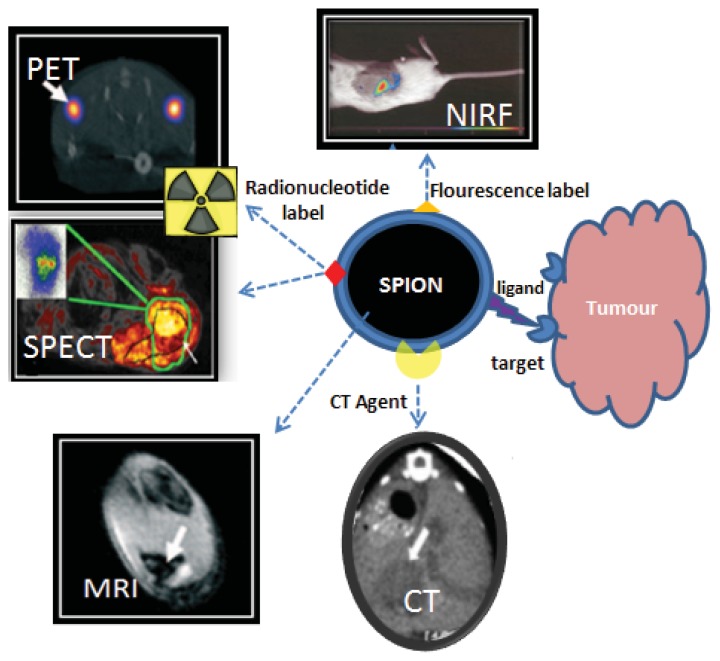
Concept of multimodal contrast agent based on SPION.

Imaging modalities vary in sensitivity, resolution, and quantitative capabilities. The major dilemma faced in single-modality imaging is the inability to assure the conformance of diagnosis, which is a crucial factor in determining the treatment. This problem can be solved by multimodal imaging because each imaging modality offers its own unique benefits. To overcome the intrinsic limitations of a specific modality, multimodal imaging offers a combination of techniques with complementary strengths [38,39]. For example, PET images provide high-sensitivity biological and functional information about cancer. Conversely, CT and MRI can offer high-resolution images to gather anatomical information. Therefore, a combination of these imaging modalities can provide not only high sensitivity and resolution simultaneously but also more detailed anatomical or biological information about cancer. Figure 1 represents a general scheme for combining different modalities into a single nanoparticle based on an iron oxide core. Multimodal imaging probes that carry more than two imaging agents have the potential to overcome the limitations of a single imaging modality. They might also provide detailed information on the target site by targeted delivery. Multimodal imaging for cancer theranostics is a cutting-edge technology that maximizes the advantages of nanoparticles [7,19]. Iron oxide nanoparticles are an attractive option as a base platform for multimodal agent development due to their intrinsic material properties that allow for tunable pharmacokinetics and surface modification.

### 4.1. MRI/Optical Imaging

The development of MRI and NIRF dye conjugated contrast agents is a major research effort in MRI/optical dual imaging strategies [40,41]. MRI is an excellent anatomical imaging technique, but it is incapable of measuring molecular events such as protease activity and gene expression. However, NIRF imaging can produce the functional details of a molecular event. Therefore, these techniques complement each other to improve overall imaging quality [42]. Among the NIRF dyes, cyanine dye (Cy5.5) is widely used in SPION. MRI and optical imaging are the most well accepted techniques in multimodal imaging and are better associated with NIRF-based molecular imaging probes with emission wavelengths ranging from 650 to 900 nm [43].

Our group fabricated MRI/optical dual-contrast agents specifically for *in vivo* cancer imaging by thermally cross-linking SPION using Si-OH containing co-polymer [44]. The optical imaging property was added by conjugating Cy5.5 dye to SPION after amine modification. The Cy5.5-labeled SPION was tested in Lewis lung carcinoma-bearing mice and resulted in a tumor specific T2 signal drop of 68% in MRI, with fluorescence lasting up to 4 h in optical imaging. Additionally, the specific accumulation of Cy5.5-SPION in the tumor was further confirmed by comparing *ex vivo* images of harvested tumor and other organs.

**Figure 2:**
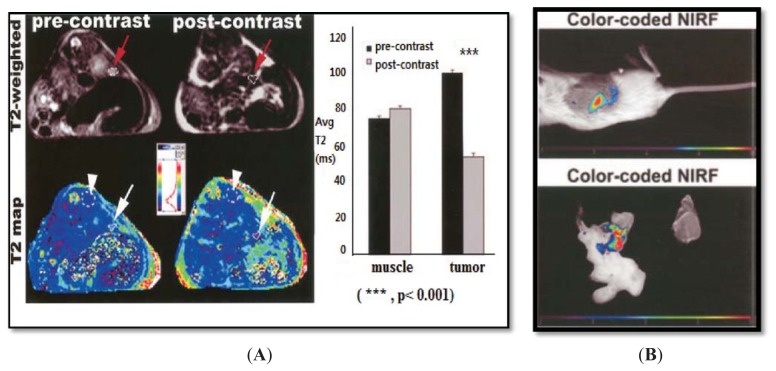
(**A**) T2 weighted images. The images are before and after the injection of CLIO-EPPT in human pancreatic adenocarcinoma. The T2 relaxation rate is 46.5% (arrow), whereas muscle (arrowhead) is not affected; (**B**) Color-coded map of NOD/SCID mice bearing orthotopically implanted human pancreatic adenocarcinoma obtained 24 h after I.V. injection of CLIO-EPPT that shows high-intensity fluorescence. Reprinted with permission from [45].

In another study performed by Medarova *et al.*, underglycosylated mucin-1 tumor-specific antigen (uMUC-1) targeted CLIO (dextran-coated crosslinked SPION) was synthesized [45]. CLIO was modified with NIRF-capable Cy5.5 dye. The EPPT peptides conjugated to CLIO can bind to uMUC-1 antigen. EPPT is a synthetic peptide derived from CDR3 V_h_ region of an adult skeletal muscle monoclonal antibody, which can specifically bind to uMUC-1 antigen. This probe could not only detect orthotopically implanted preclinical models of adenocarcinomas but could also track *in vivo* tumor response to chemotherapy in real time. T2 relaxation rate of CLIO-EPPT in the tumor dropped about 46.5% in orthotopically implanted pancreatic adenocarcinoma (Figure 2A), a change that was comparable to the 53% signal drop of the subcutaneous tumor model the group has previously studied as a proof-of-principle [46]. *In vivo* NIRF optical imaging, using CLIO-EPPT, showed high-intensity fluorescence (Figure 2B).

### 4.2. MRI/CT Imaging

Anatomical imaging is an important feature requiring the collection of information regarding lesion occurrence in the body to enable further decisions. Although both MRI and CT are relatively weak in functional imaging, they are excellent modalities for anatomical imaging. A MRI/CT dual-modal agent is a combination of two anatomic modalities that share almost the same functional features [35]. The main difference between MRI and CT is the physics involved in the respective imaging processes. CT uses X-ray radiation passing through tissue. The image is recorded based on the absorption and attenuation characteristics of the contrast agent. In contrast, MRI works under the influence of a strong magnetic field, and the image is analyzed on the basis of the T1 or T2 contrast enhancement induced by nanoparticles [47].

**Figure 3:**
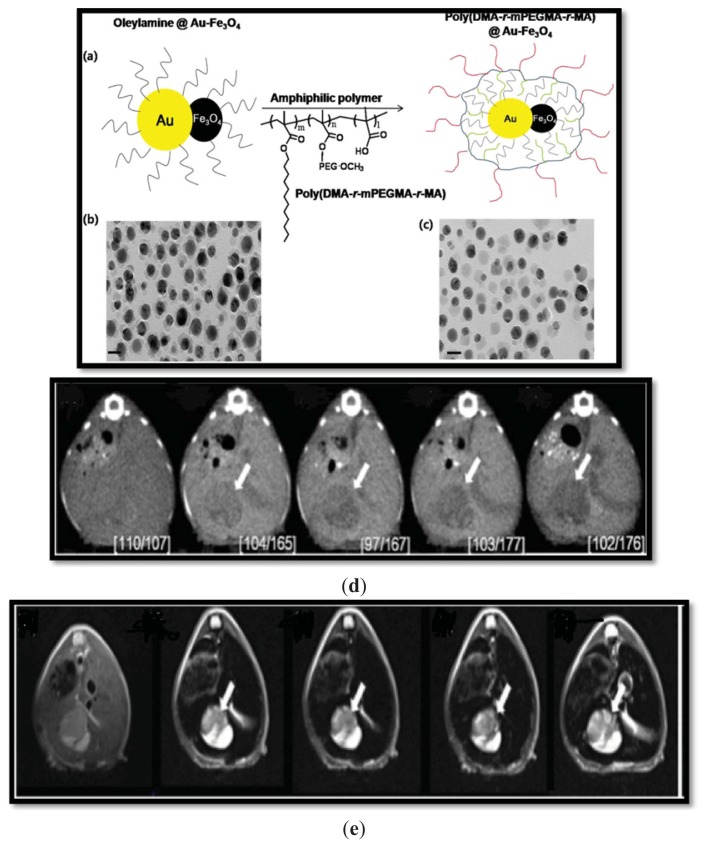
Schematic illustration (**a**) shows hybrid nanoparticles before and after coating with amphiphilic poly (DMA-r-mPEGMA-r -MA), also shown in TEM images (**b**) and (**c**), respectively. CT (**d**) and MRI (**e**) images in a hepatoma model at various time points. A white arrow indicates the site of the tumor. Reprinted with permission from [49].

Gold nanoparticles are most commonly used as CT contrast agents. They are combined with SPION to form a hybrid known as gold iron oxide nanoparticles (GION), which can be used as a dual-contrast agent for MRI and CT [48]. Gold acts as the CT contrast moiety, but it provides less spatial resolution and sensitivity compared to MRI. A dual-contrast agent with an iron oxide core and a gold-layered shell with PEG coating on the surface was synthesized to provide synergistic effects in both CT and MRI. The PEG-coated GION was dispersed in water, and its average hydrodynamic size was approximately 47 nm when measured by TEM. The intensity of the CT images for PEG coated GION was greater than iodine-based counterparts at the same concentration, but the T2 signal efficiency decreased compared to oleic acid/oleylamine coated SPION. Our group [49] reported the synthesis of hybrid nanoparticles by thermal decomposition of Au-oleylamine and Fe-oleate mixtures, followed by subsequent coating with amphiphilic poly(DMA-*r*-mPEGMA-r-MA) by nano-emulsion. The resulting nanoparticles showed acceptable water dispersion and biocompatibility (Figure 3). The *in vivo* CT contrast efficiency had improved contrast enhancement (1.6-fold) 1 h post-injection in a murine hepatoma model. The T2 relaxivity coefficient was greater than that of Resovist^®^. Additionally, the *in vivo* result was noticeably different from hepatoma and normal tissue.

The hybrid GION serves as a dual-contrast agent effectively by providing better contrast than iodine agents. The hybrid gold-SPION also displayed better CT contrast enhancement and relatively high T2 relaxivity in MRI. These two cases serve as strong examples of multimodal system development and demonstrate that multimodality is superior to a single-modal system.

### 4.3. MRI/PET Imaging

MRI/PET imaging evolved from PET/CT and is the preferred modality for anatomic and functional imaging. MRI/PET has a distinct advantage over PET/CT because of the reduced radiation exposure. PET is a nuclear imaging technique that is similar to SPECT because it is based on radionucleotide-emitting positrons. MRI obtains an image with enhanced spatial resolution, thereby correcting partial-volume effects caused by PET imaging. Multimodal PET/MRI consists of a radionucleotide conjugated with an MRI agent SPION [50].

Yang *et al.* developed a water-soluble, SPION-based nanocarrier [51]. A tumor with integrin αvβ3 expression was targeted using PEGylated SPION by cyclic Arg-Gly-Asp-D-Phe-Cys (cRGD) peptides, and dual modality imaging was attained using PET ^64^Cu chelators for PET imaging. However, the MRI relaxivity for cRGD-conjugated SPION was lower than that for clinically available iron oxide particles (Feridex^®^). PET imaging better evaluated the capability of cRGD-conjugated SPION to accumulate in tumors. The same targeting strategy was applied by Lee *et al.*, in which the αvβ3-integrin expressing tumor was targeted using iron oxide nanoparticles coated with polyaspartic acid (PASP) [52]. The RGD peptide was used as an integrin αvβ3 targeting agent, and 1,4,7,10-tetraazacyclododecane-*N*,*N*′,*N*″,-tetraacetic acid (DOTA) chelators were used after labeling with ^64^Cu for PET. DOTA IO-RGD conjugates bound specifically to integrin αvβ3 *in vitro*. MRI/PET imaging showed integrinspecific delivery with significant uptake of conjugated RGD-PASP-IO (Arg-Gly-Asp-D-Phe-Cys-Polyaspartic acid-iron oxide) nanoparticles in the reticuloendothelial system (RES). The reason stated for the significant uptake was the hydrodynamic size of the nanoparticle, which is approximately 45 nm. The particle is large enough for RES detection and uptake. Further optimization to control for the size and tumor specificity has been identified as a goal for future studies.

**Figure 4:**
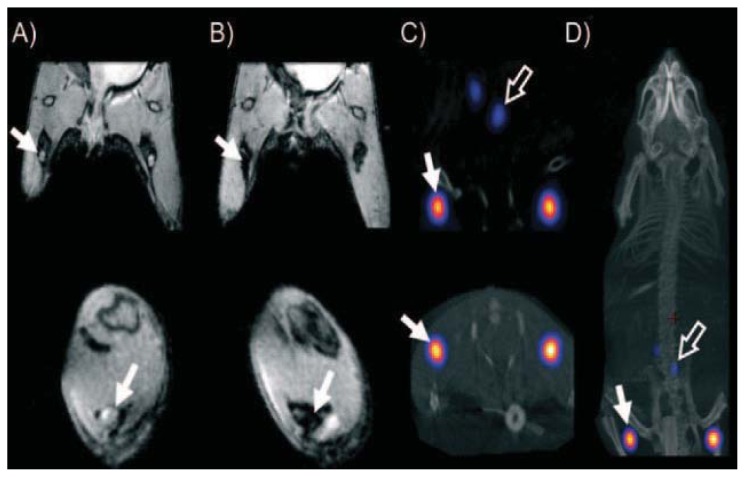
*In vivo* PET/MRI imaging studies with [^64^Cu (dtcbp) 2]–Endorem in a mouse. (**A**) and (**B**) show signal decreases in popliteal lymph nodes before and after injection indicated by solid arrows; (**C**) refers to NanoPET/CT images, showing uptake in popliteal (represented by solid arrows) and iliac (represented by hollow arrows) lymph nodes; (**D**) A whole body NanoPET–CT image of the mouse. Reprinted with permission from [53].

In another work by Torres Martin de Rosales *et al.*, ^64^Cu radiolabeling was performed directly on the inorganic surface rather than the SPION coating using bisphosphonates (BP) [53]. The chelating agent dithiocarbamate (DTC) was used for conjugating the ^64^Cu to the BP. The final complex formed [^64^Cu (dtcbp) 2] (dithiocarbamate bisphosphonates) was stable in PBS and human serum for 48 h. Then, the [^64^Cu(dtcbp)2] complex was labeled with clinically available dextran-coated SPION by virtue of BP’s high affinity to iron oxide. *In vivo* studies were conducted in a 9.4 T NMR magnet and a NanoPET–CT scanner. The *in vivo* model for the study was the lymphatic system, which provided information regarding the early spread of cancer. A foot pad injection of [^64^Cu(dtcbp)2]–endorem resulted in a notable decrease in MR signal in popliteal lymph nodes after 3 h, which provides support for endorem accumulation. PET imaging of ^64^Cu-induced signal confirmed the uptake of nanoparticles in popliteal lymph nodes and iliac lymph nodes (Figure 4).

### 4.4. MRI/SPECT Imaging

There has also been increased interest in combining SPION with SPECT probes for MRI/SPECT dual-modality imaging (Table 3). One advantage of SPECT is the opportunity to obtain information on molecular processes using specific radiolabels. SPECT also allows a clinician to determine the biodistribution of the radiotracer tagged particles *in vivo* non-invasively in the pico-molar concentration range. However, a disadvantage of SPECT is that it offers limited anatomical details and spatial resolution. MRI is used in conjunction with SPECT to obtain quality anatomical images, thus offering both the structural and functional benefits of dynamic imaging. MRI/SPECT reduces discomfort associated with multiple sessions and scan times required for separate imaging procedures [4].

**Figure 5:**
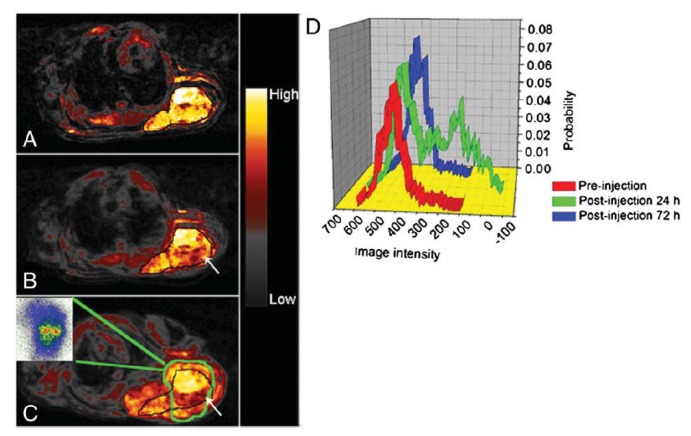
T2-weighted axial MRI for A431K5 tumors. (**A**), (**B**), and (**C**) show pre-injection and 24 and 72 h post-injection MR images. The inset represents autoradiographic images of 20 μM tumor sections; (**D**) ROI intensity histograms for T2-weighted axial gradient-echo MR images of A431K5 tumors, which show apparent shifts in a 24-h image towards low intensity. Reprinted with permission from [4].

Recently, Misri and colleagues have developed an antibody-conjugated MRI/SPECT dual-modality imaging probe specifically for malignant mesothelioma [4]. Mesothelin targets antigens for malignant mesothelioma using ^111^In labeled anti-mesothelin monoclonal antibody (mAbMB) coated on iron oxide nanoparticles. A cell uptake study showed specific uptake of In-mAbMB-SPION by mesothelin-positive cells. An *in vivo* study of a mouse xenograft model of A431K5 tumors showed a signal drop post-24-h scan. The biodistribution study using SPECT imaging also showed relatively low uptake in the other normal organs (lungs, heart, intestine, stomach, muscle, and brain) compared to the tumor. This result was well correlated with autoradiography images (Figure 5).

In another example, Madru *et al.* developed ^99m^Tc-labeled PEG coated iron oxide as a multimodal contrast agent for imaging a sentinel lymph node (SLN) [55]. The SLN is considered the first lymph node receiving the lymphatic drainage from a malignant tumor, where metastatic cells might anchor initially. MRI/SPECT both aided in identifying the SLN and provided pre-surgical information regarding the location and characteristics of the lymph node. SPECT gave a clear visualization of the lymph node and the highest intensity was found in the popliteal lymph nodes. The clearance of ^99m^Tc-SPION was comparable to the other similar sized nanocolloids. The MR image showed a non-homogenous uptake of ^99m^Tc-SPION. This approach can be used for breast cancer and malignant melanoma imaging.

The dual-modal probe developed by Misri and colleagues displayed better MR relaxivity compared to previously developed MRI/SPECT agents. The targeting efficiency with mesothelin-positive tumors was proven, and the SPECT detection motif ^111^In was used to obtain autoradiography and biodistribution data. In the work of Madru *et al.*, the radiolabeling efficiency of ^99m^Tc-labeled PEG coated iron oxide reached 99%. The SLN uptake of nanoparticles was 100% ID/g, suggesting that the nanoparticle efficiency and SPECT/MRI detection in lymph node should encourage its usage in other cancers.

## 5. Multifunctional Magnetic Nanoparticle for Cancer Theranostics

**Figure 6:**
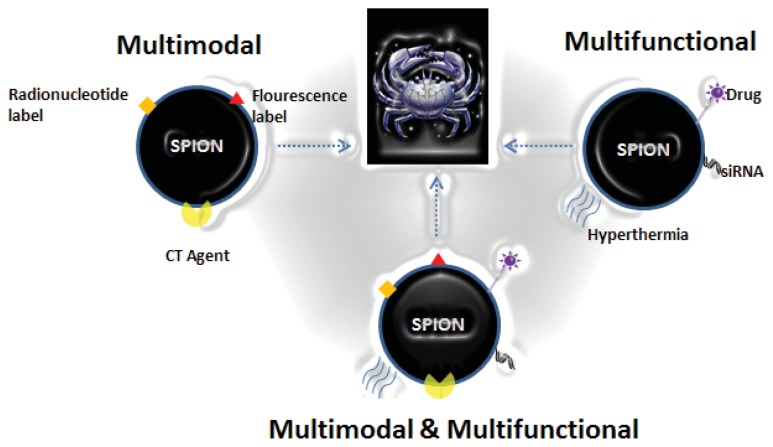
Different approaches adopted in developing a SPION-based theranostic agent.

The multi-functionality of nanoparticles further enables the integration of imaging and therapy (so-called theranostics). Theranostics refer to agents that target molecular biomarkers of a disease and are expected to contribute to personalized medicine. Among the currently available nano-vehicles, SPION have received great attention in the development of theranostic nanomedicines because they are not only used as contrast enhancement agents for MRI but can also deliver therapeutic agents, such as anticancer drugs and siRNA, to disease sites. In addition, SPION can continuously emit heat upon exposure to an alternating external magnetic field (AMF) by converting electromagnetic energy into heat [56–61]. Figure 6 depicts three different approaches in developing a SPION-based theranostic agent.

### 5.1. Drug Delivery

Chemotherapy is a common therapeutic approach to fighting cancer. However, it is non-selective and causes significant potential side effects to healthy tissues. To overcome this problem, magnetic nanoparticles loaded with the drug can serve as potential drug carriers in a new drug delivery strategy (Table 4). The targeted delivery of therapeutics has the potential to localize therapeutic agents to specific tissues to enhance treatment efficacy and minimize side effects. Recently, several chemical drugs, including paclitaxel, doxorubicin (DOX), and methotrexate (MTX), have been combined with magnetic nanoparticles for cancer therapy [40,56,62].

The multi-functionality of a SPION-based nanosystem was explored using drug and imaging modalities. With the development of biocompatible polymer-coated SPION loaded with DOX, Yu *et al.* [62] successfully evaluated its tumor-reduction efficacy in lung cancer. Additionally, imaging from MRI and NIRF provided diagnostic information. Santra [56] developed a theranostic nanoparticle with similar MRI/optical capabilities and a folate receptor-targeting moiety. The A549 lung cancer cell line was used to evaluate cell uptake and study toxicity. A high tumor-specific uptake and doxorubicin-induced cell death were observed. Another unique triple-modal system developed by Xie *et al.*, was modified in an adjunct study to include a DOX in the HSA (Human Serum Albumin) matrix to form DOX conjugated HSA–iron oxide nanoparticles [62]. The evaluation of tumor reduction in 4T1 tumor-bearing mice revealed that the DOX conjugated HSA–iron oxide nanoparticle was superior to DOX and similar to Doxil.

**Figure 7:**
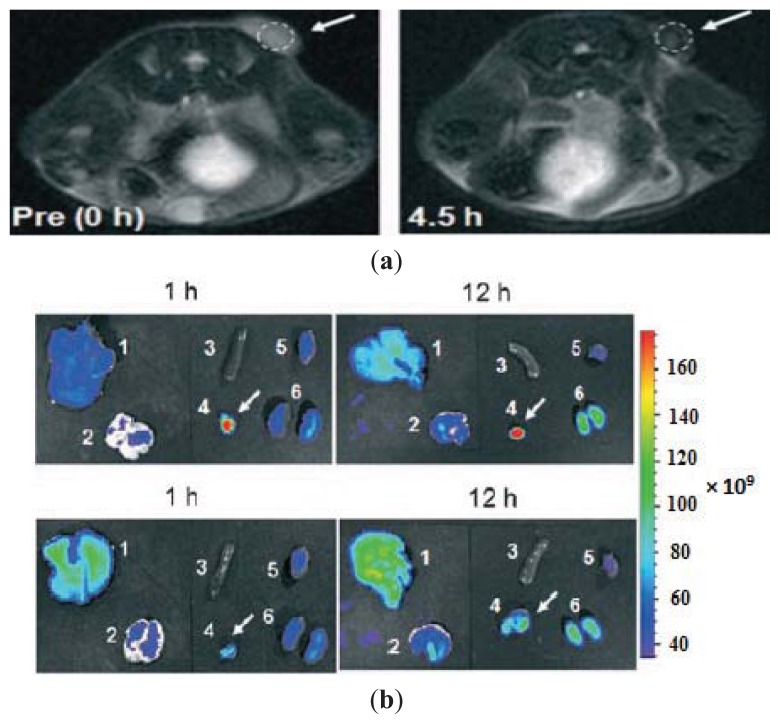
(**a**) T2-weighted MR images taken at 0 h and 4.5 h after injection of Dox@TCL-SPION at the LLC tumor on the right back of the mouse; (**b**) Optical fluorescence images of major organs and allograft tumors: 1 liver; 2 lung; 3 spleen; 4 tumor; 5 heart; and 6 kidney. Images were taken after intravenous injection of Dox@TCL-SPION (equivalent to 4 mg of Dox) (above) and free Dox (4 mg) into tumor-bearing mice (below) (*n* = 3). The mice were euthanized after 1 h and 12 h. Reprinted with permission from [63].

Yu *et al.* [63] synthesized thermally cross-linked SPION (TCL-SPION) and coated them with a negatively charged anti-biofouling polymer and Cy5.5 dye. The nanoparticles were then loaded with positively charged doxorubicin (DOX) using electrostatic interactions. The nanoparticles were systematically injected into lung cancer bearing mice and resulted in a significant decrease in tumor size. The nanoparticle uptake in this study was achieved by the EPR effect using a passive targeting strategy and was evaluated by *in vivo* MRI and optical imaging (Figure 7). Dox-TCL-SPION were injected in mice with Lewis lung carcinoma. The data indicate that the tumor reduction was approximately 63% relative to control and 38% for the DOX group. A T2-weighted image of mice suggested a 58% drop in signal, indicating significant accumulation in the tumor. An *ex vivo* fluorescence study showed intense signal at the tumor area 1 h post-administration and maximum intensity at 12 h post-injection.

Santra *et al.* developed a theranostic nanoparticle with MRI/optical functionality and targeted cancer cells expressing folate [56]. A co-encapsulation strategy was adopted to carry a NIR dye and an anti-cancer drug in the hydrophobic region of polyacrylic acid (PAA), which is coated on SPION by a modified solvent diffusion technique. Initially, iron oxide coated with PAA formed propargylated (presence of triple-bond) nanoparticles that served as a precursor for multimodal folate conjugated nanoparticles by click chemistry. The versatility of the nanosystem was proven by the encapsulation of taxol and lipophilic fluorescent dye in the hydrophobic region. The feasibility of the nanoparticles for use in *in vivo* studies was confirmed by encapsulating NIR dialkylcarbocyanine dye and tested for *in vitro* uptake in an A549 lung cancer cell line. The fluorescence measurement demonstrated higher cell uptake for NIR and folate receptor compared with the control group. The cell study showed targeted uptake of nanoparticles in A549 cells with induction of cell death mediated by the acidic nature of a tumor cell, which assisted in the release of dye and taxol. Finally, modifying the targeting ligand can customize the ligand for other cancer types.

### 5.2. Gene Delivery

Gene therapy entails the delivery of a therapeutic gene to the disease tissue to functionally replace a defective gene and cure the pathological genotypes by expression of a therapeutic gene. Small-interfering RNA (siRNA) is a class of double-stranded RNA molecules that can inhibit any specific protein expression at the post-transcriptional level. The process is known as RNA interference (RNAi). Genes are integrated into SPION, which protect the nucleic acids against enzymatic degradation and facilitate cellular internalization and endosomal release (Table 5). SPION are believed to be an excellent vehicle for siRNA delivery because they are biocompatible and target-functionalized [65]. Even hard-to-transfect cell lines can be delivered with plasmid DNA or siRNA by novel methods such as magnetofection, where SPION is subjected to oscillating magnetic fields that facilitate caveolae-mediated endocytosis of SPION and cargo nucleic acid [66,67]. MRI-visible SPION have also been found to carry a dual-modality function that enhances monitoring of siRNA delivery to the target area [68].

SPION functionalized with PEG grafted polyethylenimine (PEG-g-PEI) was synthesized by Chen *et al.* using the CD44v6 single-chain variable fragment as a targeting agent for gastric cancer, and siRNA was loaded for the therapy [69]. Compared to viral vector delivery, non-viral vector PEG-g-PEI had less cytotoxicity and consisted of primary, secondary, and tertiary amines for forming complexes with siRNA. The strategy used single-chain variable fragment (scFv) targeting CD44 variant 6 (CD44v6) antigens. The transfection efficiency was studied using flow cytometry against lipofectamine (a control siRNA delivery agent in SGC-7901 gastric cancer cells). The results showed an efficiency of more than 95% for all three siRNA delivery agents, and fluorescence intensity tests revealed that the scFvCD44v6-PEG-g-PEI-SPION complex intake was greater than that of the PEG-g-PEI-SPION complex. To assess cancer-targeting capability, two tumors (SGC-7901 and A375), one CD44v6 positive and the other negative, were induced in mice, and PEG-g-PEI-SPION and scFvCD44v6-PEG-g-PEI-SPION contrast agents were injected. As expected, the SGC-7901 tumor area showed a T2 signal drop for the scFvCD44v6-PEG-g-PEI-SPION group and proved targeting capability.

An all-in-one nanoparticle was developed with multimodal imaging and siRNA delivery capability by Lee *et al.*, in which the fabricated core material was manganese-doped magnetism-engineered iron oxide (MnMEIO) nanoparticles coated with BSA [61]. This nanoparticle was selected for use in MRI applications because of its small particle size (15 nm), monodispersed nature and higher magnetic moment than other iron oxide nanoparticle variants. To conjugate the siRNA and thiolated siRNA (HS–siGFP–Cy5) targeting agents onto the surface of the nanoparticle, BSA was modified with pyridyldisulfide groups by *N*-succinimidyl-3-(2-pyridyldithio)propionate (SPDP) treatment. Thus, activated MnMEIO nanoparticles were treated and functionalized with thiolated poly(ethylene glycol) (PEG, MW 3400), cyclic Arg-Gly-Asp (RGD) peptide and Cy5-dye labeled thiolated siRNA (HS–siGFP–Cy5). The effect of MnMEIO–siGFP–Cy5/PEG–RGD nanoparticles was studied in cell lines expressing GFP (MDA-MB-435-GFP and A549-GFP cells), and the result showed dose-dependent decreases in GFP expression, which validate the effect of synthesized nanoparticles.

### 5.3. Hyperthermia

Hyperthermia is a form of treatment in which the temperature of body tissue is raised to 42 °C. This treatment drastically affects the functionality of cellular structures, proteins, cell membrane, nucleic acid repair enzymes, and induces cell death. Tumor cells can also be killed due to their lower tolerance of a sudden variation in temperature using magnetic hyperthermia (MHT). SPION possess the inherent property of generating heat by ferromagnetic resonance. However, the heating process is generated by Néel and Brownian relaxations during MHT treatment [58]. Therefore, it has been widely studied for treatment in different types of cancer, such as head and neck cancer, glioma, and cervical cancer [59,60].

The possibility of using SPION in both multimodal and nanoheater applications has been studied by Hoskins *et al.* [70]. SPION were synthesized with an average size of 30 nm in diameter and coated with gold and PEI as the intermediate layer. Finally, PEG was coated on the particles to form Fe_3_O_4_-PEI-Au-PEG nanoparticles with improved biocompatibility. To study the hyperthermia effect, the nanoparticles were suspended in agar to mimic *in vivo* conditions. Applying laser irradiation at a 532-nm wavelength for 90 s to a nanoparticle concentration of 50 μg/mL induced changes in temperature of approximately 32 °C. The MRI contrast effect was found to be comparable to that of Feridex^®^. PEI acts as a cushion between the core and shell and helps to maintain their physical properties. The magnetic properties of SPION had also been retained after the gold coating was applied. This study raises the possibility of using SPION-GOLD hybrid nanoparticles for multimodal contrast imaging and cancer treatment by hyperthermia.

## 6. Conclusions

Multimodal and multifunctional probes are cutting-edge technologies in which the advantages of nanoparticles are maximized. The use of MRI has prompted the development of a variety of multimodal probes based on T2 contrast agents. SPION is considered an ideal vehicle for multimodal and multifunctional applications. The theranostic agents based on SPION are crucial in cancer detection and the delivery of therapeutic payloads, such as chemotherapeutic drugs and genes. New research is dedicated to developing multifunctional and multimodal theranostic agents, which form the ultimate weapon against cancer. However, there are several challenges facing multimodal and multifunctional techniques, including the standardization of therapy response and the stability of complex nanoparticles under certain biological conditions. Future research must focus on rectifying the current flaws in multimodal and multifunctional imaging probes by introducing new biocompatible polymers. Finally, multimodal and multifunctional techniques may enhance clinical theranostics in the near future.

## Figures and Tables

**Table 1:** Comparison of synthesis methods of magnetic nanoparticles. Modified with permission from [26].

Method	Parameters	Reaction temperature (°C)	Reaction time	Solvent	Size (nm)	Yield
Thermal decomposition	Nitrogen atmosphere, reflux condition	100–320	Slow	Water	1.5–8	High
Co-precipitation	Ambient condition	20–90	Fast	Organic	2–15	High
Microemulsion	Ambient condition	20–50	Fast	Organic	2–12	Low

**Table 2:** Advantages and disadvantages of imaging modalities. Reprinted with permission from [28].

Modality	Advantages	Disadvantages
MRI	High spatial resolution	Low sensitivity
	Good soft tissue contrast	Relatively long acquisition time
	Provides both anatomical and functional information	Requires expensive equipment
PET	Provides biochemical information	Limited anatomical information
	High sensitivity	Requires specialized equipment
	Three-dimensional imaging	Requires radio-nucleotide facilities
	Can monitor changes in tumour metabolism and drug biodistribution	Requires expensive equipment
SPECT	Potential to detect multiple probes simultaneously in contrast to PET	Lower sensitivity than PET
		Lower resolution
CT	High-sensitivity anatomical imaging	Limited functional information
	Provides three-dimensional image	Poor soft tissue contrast
		Requires expensive equipment
Optical (BLI and fluorescent)	Wide applicability	Requires genetic manipulation of investigated cells
	Simultaneously monitor several molecular events	Provides limited anatomical information
	Relatively inexpensive	
	Amenable to smaller research laboratories	Reduced sensitivity with increased imaging depth

**Table 3:** Characteristics of nanoparticles for multimodal imaging.

Imaging modality	Types of nanoparticle	Pros	Cons	References
MRI/Optical	Cy5.5-SPIONCy5.5-CLIO-EPPT	Good anatomical detailing, and functional detailing of molecular event	Passive targeting	[44,45,54]
MRI/CT	GION, Hybrid Gold SPION	Good anatomical detailing	Poor functional imaging capability No tumor targeting	[48,49]
MRI/PET	^64^Cu -crude-SPION, ^64^Cu -PASP-IO	High sensitivity	Different dosage of PET and MRI agents.	[51–53]
MRI/SPECT	^111^ In -Bam-IO,^99m^Tc-PEG-IO,99mTc-DPA-SPION	High sensitivity and functional information	-	[4,5,55]

**Table 4:** Characteristics of multifunctional magnetic nanoparticles in drug delivery.

Nanoparticle property	Modality	Drug	References
cy5.5-SPION	MRI/Optical	Doxorubicin	[63]
PAA-IONPs	MRI/Optical	Taxol	[56]
^64^Cu-Cy5.5-HSA-SPION	PET/NIRF/MRI	Doxorubicin	[62,64]

**Table 5:** Characteristics of multifunctional magnetic nanoparticles in gene delivery.

Nanoparticle property	Modality	siRNA action	Transfection efficiency	References
scFvCD44v6,-PEG-g-PEI-SPION	MRI	Not studied	95%	[69]
MnMEIO–siGFP–Cy5/PEG–RGD	MRI/Optical	GFP Suppression	Not studied	[61]
